# A Systematic Comparison of Protocols for Recovery of High-Quality RNA from Human Islets Extracted by Laser Capture Microdissection

**DOI:** 10.3390/biom11050625

**Published:** 2021-04-22

**Authors:** Chiara M. A. Cefalo, Teresa Mezza, Andrea Giaccari, Rohit N. Kulkarni

**Affiliations:** 1 Islet Cell Biology & Regenerative Medicine, Joslin Diabetes Center, Department of Medicine, Brigham and Women’s Hospital, Harvard Medical School, Harvard Stem Cell Institute, Boston, MA 02215, USA; cefalo.chiara@gmail.com (C.M.A.C.); teresa.mezza@gmail.com (T.M.); 2 Dipartimento di Scienze Mediche e Chirurgiche, Centro per le Malattie Endocrino-Metaboliche, Fondazione Policlinico Universitario Agostino Gemelli IRCCS, 00168 Rome, Italy; 3 Dipartimento di Medicina e Chirurgia Traslazionale, Università Cattolica del Sacro Cuore, 00168 Rome, Italy

**Keywords:** laser capture microdissection, RNA extraction, human islets

## Abstract

The isolation of high-quality RNA from endocrine pancreas sections represents a considerable challenge largely due to the high ribonuclease levels. Laser capture microdissection (LCM) of mammalian islets, in association with RNA extraction protocols, has emerged as a feasible approach to characterizing their genetic and proteomic profiles. However, a validated protocol to obtain high-quality RNA from LCM-derived human pancreas specimens that is appropriate for next-generation sequencing analysis is still lacking. In this study, we applied four methods (Picopure extraction kit, Qiazol protocol, Qiazol + Clean-up kit, and RNeasy Microkit + Carrier) to extract RNA from human islets obtained from both non-diabetic individuals and patients with type 2 diabetes who had undergone partial pancreatectomy, as well as handpicked islets from both non-diabetic and diabetic organ donors. The yield and purity of total RNA were determined by 260/280 absorbance using Nanodrop 100 and the RNA integrity number with a bioanalyzer. The results indicated that among the four methods, the RNeasy MicroKit + Carrier (Qiagen) provides the highest yield and purity.

## 1. Introduction

Laser capture microdissection (LCM) has emerged as a widely used technique for the isolation of specific types of cells or a minimum amount of parenchyma [1] for a variety of downstream analyses such as proteomic studies [2,3], RNA assays by microarray [4], or RNA sequencing [5,6]. This approach has been particularly useful to obtain small amounts of islet tissue to characterize the genetic profiles of both murine and human pancreas [7]. Compared to the analyses of handpicked islets, the use of LCM with specific staining for target cells avoids contamination by neighboring cells and the confounding effects of cell trauma/ischemia, which leads to alterations in cellular protein and gene expression due to the harsh chemical and/or mechanical processes during manual isolation. Indeed, previous reports [8], using handpicked islets, show an upregulation of pancreatic acinar and duct genes, suggesting contamination by non-endocrine cells, while the elevated expression of hypoxia- and apoptosis-related genes indicates changes secondary to mechanical effects. These findings were confirmed by Paraskevas et al. [9], who reported an upregulation of inflammatory markers, such as cytokines and cytokine receptors, in freshly isolated islets compared to beta cells from intact pancreas collected by LCM. Moreover, freshly isolated cells cultured for three days displayed a greater expression of transcription factors found in pancreatic progenitors, suggesting that islet isolation and culture together activate a process of de-differentiation of endocrine pancreatic cells. Thus, using chemical approaches to isolate pancreas islets could misrepresent the gene expression profile.

Given the limitations of manual islet isolation, the LCM approach is preferable to limit confounding results. Nevertheless, harvesting high-quality RNA from human islets that can be used for transcriptomic analysis is a continuing challenge, mostly due to the high level of intrinsic ribonuclease (RNase) activity in the pancreas [10,11]. One possible approach to minimizing the effect of RNase activity on the isolated cells and to increase RNA quality is the addition of a RNase inhibitor during both LCM and RNA extraction phases, as previously reported by Butler et al. [12]

Although the LCM technique [13] has improved, the quality and quantity of RNA collected from LCM human pancreatic samples is generally low, with no more than 100 ng of material after RNA amplification, compared to the 200–500 ng of RNA with at least an RNA integrity number (RIN) of 7, necessary to generate cDNA libraries for gene expression [14]. In this report, we compare the efficiency of different protocols used to extract RNA from human islet samples obtained by LCM in order to determine a better approach to ensuring RNA preservation that can be used by the scientific community for next-generation transcriptomic studies.

## 2. List of Equipment and Reagents

The following reagents were used:

- Tissue-Tek OCT medium (Sakura Finetek, Flemingweg, NL, USA, Cat# 4583)
- Isopentane (2-methylbutane) (Fisher Scientific, Waltham, MA, USA, Cat# 03551-4)
- DEPC-treated water (Invitrogen, Carlsbad, CA, USA, Cat# 750024)
- Ethanol 100% (Pharmco, Brookfield, CT, USA Cat# 1000200SG)
- Ethanol 70% (dilute 100% ethanol with DEPC-treated water to obtain 70% ethanol solution)
- Xylene (Fisher Scientific, Waltham, MA, USA, Cat# UN1307)
- SUPERase·IN (Ambion, Austin, TX, USA, Cat# AM2694)
- RNeasy Micro Kit 50 (Qiagen, Germantown, MD, USA, Cat# 740049)
- Qiazol lysis reagent, 50 mL (Qiagen, Germantown, MD, USA, Cat#55402828)
- RNeasyMinElute Clean-up Kit 50 (Qiagen, Germantown, MD, USA, Cat#74204)
- PicoPure RNA isolation kit (Applied Biosystems by Thermo Fisher Scientific, Vilnius, Lithuania, Cat# KIT0204)
- RNase-Free DNase Set 50 (Qiagen, Germantown, MD, USA, Cat# 79254)

The following laboratory materials and equipment were required:

- Cryomold (Fisher Scientific, Waltham, MA, USA, Cat# 22-038217)
- Frosted microscope slides (Corning, New York, NY, USA, Cat# 2948-75X25)
- Polypropylene Falcon Tube (Fisher Scientific, Waltham, MA, USA, Cat# 14-959-49A)
- RNaseZap, 250 mL (Ambion, Austin, TX, USA, Cat# 9780)
- CapSure HS LCM Caps (Arcturus Engineering, Mountain View, CA, Cat# LCM0214)
- GeneAmp^®^ Autoclaved Thin-Walled Reaction Tubes (Applied Biosystems by Thermo Fisher Scientific, Vilnius, Lithuania, Cat# N801-0611)
- Tweezers and forceps
- Pipettes: 20–200 µL and nuclease-free pipette tips
- Cryostat
- Fume hood
- PixCell^®^ IIe Laser Capture Microdissection System (Arcturus Engineering, Mountain View, CA, USA)
- Incubator (Fisher Scientific, Waltham, MA, USA, Cat# 11690506D)
- Microcentrifuge (Fisher Scientific, Waltham, MA, USA, Cat# 05-090-128)

## 3. Materials and Methods

To compare the efficiency of different RNA extraction protocols in frozen human islet samples, we used LCM to collect a mean of 100 islets from pancreatic surgical specimens, obtained from non-diabetic or diabetic patients who had undergone partial pancreatectomy for an extra-pancreatic tumor. Surgical procedures were performed by the Hepato-Biliary Surgery Unit of the Department of Surgery (Agostino Gemelli University Hospital, Rome, Italy). All the patients involved in the study provided informed consent for tissue analyses prior to surgery.

The pancreas samples were collected from the downstream edge of the surgical cut. On the basis of 2 h glycemia after a standard glucose load (75 g) prior to surgery, patients were divided into non-diabetic (*n* = 7) or diabetic (*n* = 2), according to the American Diabetes Association guidelines [15]. Clinical and metabolic characteristics of the two groups before surgery are summarized in Table 1. As expected, patients with diabetes exhibited higher fasting blood glucose and postprandial blood glucose levels (2 h after an oral glucose tolerance test) compared to non-diabetic subjects. However, even though median HbA1c levels were higher in diabetic subjects compared to others, the difference did not reach statistical significance, suggesting the diabetes was either well compensated or the disease was in an early phase. No differences were observed in serum insulin levels or insulin resistance parameters (e.g., Matsuda index) or in the lipid profile between the two groups.

The total amount of RNA from each microdissected sample was evaluated with Nanodrop by absorbance at 260 and 280 nm; subsequently, 1 µL of RNA was analyzed by the Agilent 2100 Bioanalyzer to assess RNA quality.

To exclude potential differences due to islet sources and isolation procedures, we also performed protocols using handpicked islets from organ donors (OD islets), including one non-diabetic and one diabetic individual, provided by the Integrated Islet Distribution Program (IIDP), as positive controls.

### 3.1. Sample Processing and Sectioning

Sample processing was performed in the Endocrinology Department of the Catholic University (Rome) and shipped to the Joslin Diabetes Center (Boston) for sectioning and LCM procedures.

To maintain tissue integrity, tissue processing was completed within 60 min of the surgical removal of the tumor. The dissected pancreatic tissue specimens (1 × 1 cm^2^) were quickly placed into a cryomold and covered with an optimal cutting temperature (OCT) compound. The mold was then placed in an isopentane–dry ice bath until the OCT compound became completely solid. Finally, the frozen specimen was stored in foil in a −80 °C freezer until sectioning.

Tissue sections were cut using a cryostat maintained at −20 °C using a blade previously wiped down with 100% ethanol, and individual sections were mounted onto frosted slides. The cryoblocks were previously placed inside the cryostat for ~30 min to reach cutting temperature and avoid breakage; the cryostat’s wheel was rotated slowly and steadily to avoid the formation of bubbles or folds. At least 30 sections were processed, each of 8 µm thickness, to obtain 100 islets for each subject. Subsequently, all sections were stored at −80 °C. One slide was chosen from every five sequential sections for staining with hematoxylin and eosin to localize the islets prior to LCM sessions.

### 3.2. Laser Capture Microdissection

Islet preparations for downstream analyses were obtained from frozen samples by LCM. Prior to the LCM procedure, the sections were dehydrated and cleared in a fume hood by systematically dipping the slides one after the other in the solutions listed below:

- Rinsing in diethylpyrocarbonate (DEPC)-treated water
- 70% ethanol for 30 s
- 100% ethanol twice for 1 min
- Xylene for 4 min

All the solutions, except xylene, were prepared the day before and stored at 4 °C to ensure optimum performance. The slides were dried in the fume hood for 3–5 min before LCM. We used desiccant cartridges with silica gel (Fisher Scientific 08-594-14B) and a vacuum desiccator (Fischer Scientific F420120000) as alternative methods to more effectively dry the slides, if necessary.

LCM was performed using the PixCell II Laser Capture Microdissection System (Arcturus Engineering, Mountain View, CA, USA). The power setting for the laser pulse was at 35 mW with a duration of 2.5 ms to limit tissue trauma and provide greater precision. Subsequently, selected populations of cells were cut with the laser and mounted on transparent LCM caps provided by Arcturus. To collect material for RNA analysis, at the end of the section, the thermoplastic film containing the microdissected cells was covered with a GeneAmp microfuge tube filled with 11 µL of RNA extraction buffer (guanidine isothiocyanate and polyethylene glycol octylphenol) plus 0.5 µL of RNase inhibitor 1 U/µL (SUPERase·IN Ambion, Cat# AM2694), as previously reported [12], and incubated for 30 min at 42 °C. Each session lasted no longer than 30 min to avoid RNA degradation. After incubation, the tube and the attached cap with captured islets were centrifuged at 800 g for 1 min to collect the extract into the microfuge tube and immediately transported on dry ice and stored at −80 °C.

To avoid contamination and RNA degradation, we noted that it is necessary to accurately follow all the steps, especially during the final collection. In particular, we observed that the use of 100% ethanol and RnaseZap to clean the bench and microscope surfaces, pipettes, and forceps was critical. Other key precautions included using disposable gloves and wearing a mask during all procedures. Faster execution of LCM procedures vastly improved the quality of the RNA. Further, to minimize exocrine contamination, we selectively collected cells from the islet core.

### 3.3. RNA Extraction: Methods

After collection of islets by LCM from surgical specimens of pancreatectomized patients (PP islets), the RNA was extracted using four different protocols, whose efficacy was evaluated by comparing Nanodrop and bioanalyzer results.

1. PicoPure Extraction Kit: First, we used the PicoPure extraction kit by Applied Biosystems by Thermo Fisher Scientific, Vilnius, Lithuania), using a 1:1 ratio of extraction buffer (guanidine isothiocianate) to 70% ethanol. We included DNase treatment by incubating extracted RNA with RNase-free DNase reagent (Qiagen, Germantown, MD, USA) for 15 min. Subsequently, to obtain better RNA quality compared to previous reports (e.g., mean RIN of 5.8) [13], we added a purification step using the RNeasy Mini Clean-up kit (Qiagen, Germantown, MD, USA), which allows the concentration of at least 100 μg of total RNA (≥200 nucleotides) in an elution volume of 30–100 µL. We performed this process on islets extracted from 7 non-diabetic PP samples and 2 diabetic PP samples.
  To improve both the quantity and the quality of the RNA, we first performed RNA extraction on LCM-collected islets from one non-diabetic and one diabetic subject using Qiazol reagent as lysis cell buffer, which has been reported to produce high-quality RNA from rat pancreas [16]. We used samples that showed a better RIN after bioanalyzer evaluation following the purification step with the RNeasy Mini Clean-up kit (Qiagen Germantown, MD, USA).
  Then, to optimize the use of the extremely limited material, we performed LCM on all samples and pooled all material obtained from non-diabetic (*n* = 7) and diabetic (*n* = 2) subjects; finally, we optimized the process comparing the total amount and the integrity of the RNA extracted from the two samples with three other protocols.
2. Qiazol: In this protocol, we avoided the use of binding columns to minimize the loss of material; thus, 700 µL of Qiazol lysis reagent (Qiagen, Germantown, MD, USA) was added to each sample to permit dissociation and homogenization of nucleoprotein complexes, followed by transfer of the supernatant to a new tube. Addition of 140 µL of chloroform followed by a 15 min centrifugation step allowed the separation of the colorless aqueous upper phase containing the ribonucleic acid from the pink lower phase rich in organic proteins and included the interphase where DNA was present. Total RNA was precipitated in a gel-like pellet on the sides and bottom of the tube by mixing the aqueous phase with 350 µL of isopropyl alcohol. To avoid DNA contamination, we incubated the extracted material for 15 min with RNase-free DNase reagent (Qiagen, Germantown, MD, USA). Two washing steps with ethanol were performed to remove contamination, and the extracted RNA was dissolved in 40 µL of DEPC-treated water for downstream analysis.
3. Qiazol/Clean-up: In testing this method, we used the previous protocol that included a purification step using the RNeasy Mini Clean-up kit (Qiagen, Germantown, MD, USA, Cat#74204), according to the protocol suggested by the manufacturer (Qiagen). The use of a mini spin column allowed binding of total RNA to the membrane, while the contaminants were efficiently washed away. The final RNA was dissolved in 14 µL of DEPC-treated water.
4. Microkit/Carrier: In this protocol, we used Qiazol as lysis cell buffer and performed RNA extraction using the RNeasy Microkit 50 (Qiagen, Germantown, MD, USA, Cat# 740049). This method was designed for isolation of total RNA (up to 45 µg) from small samples. To improve the recovery of total RNA from small samples, we added 5 μL of a 4 ng/μL working solution of poly-ARNA carrier to the lysate. Subsequently, the first steps of the column-based isolation protocol were the same as the first method tested (PicoPure extraction kit by Applied Biosystems by Thermo Fisher Scientific, Vilnius, Lithuania) and involved Qiazol and chloroform solutions to lyse and homogenize samples. Ethanol was added to reach ideal binding conditions, and the lysate contained in the aqueous phase was transferred into the RNeasy MinElute spin column to allow RNA binding to the silica membrane. DNase and any contaminants were efficiently washed away with ethanol, and pure concentrated RNA was eluted in 14 µL of DEPC-treated water.

In all the protocols described above, we added 0.75 μL of RNase inhibitor to the total volume of isolated RNA at the end of each procedure to minimize its degradation.

## 4. RNA Extraction: Results

Nanodrop assessment of RNA, extracted from both non-diabetic and diabetic PP islets using the first protocol (PicoPure extraction kit by Applied Biosystems by Thermo Fisher Scientific, Vilnius, Lithuania) and performed before and after the purification experiment, is reported in Table 2.

**Figure 1:**
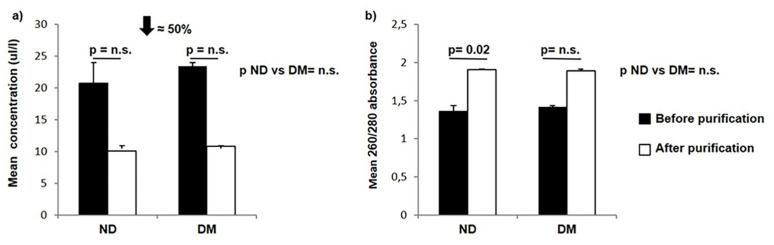
Concentration (**a**) and 260/280 absorbance (**b**) of RNA extracted with the Arcturus PicoPure extraction kit before (black bars) and after (white bars) the purification step with the RNeasy Mini Clean-up kit in PP islets of non-diabetic and diabetic subjects. ND: non-diabetic PP; DM: diabetic PP, value interaction between pre- and post-purification process. A 50% reduction in the mean concentration was evident in both groups after purification. Variables are expressed as the mean ± SD. PP: pancreatectomized patients.

We observed a 50% loss of RNA after the purification step in both non-diabetic and diabetic subjects (Figure 1a) despite the improvement in the 260/280 absorbance in both groups, which reached statistical significance only in the non-diabetic group (Figure 1b). RNA concentrations were comparable between non-diabetic and diabetic patients both before and after purification (before purification *p* = 0.83; after purification *p* = 0.88) (Figure 1b). The mean 260/280 absorbance showed no significant difference between non-diabetic and diabetic subjects in both cases (before purification *p* = 0.7; after purification *p* = 0.92) (Figure 1b).

Although we observed relatively good 260/280 ratios, as assessed by Nanodrop after the purification step (Table 2), the bioanalyzer evaluation showed a lower concentration and a low RNA integrity number, suggesting degradation of RNA (Table 3).

Nanodrop assays for the RNA extraction protocols performed using Qiazol as lysis buffer on PP islets of one non-diabetic sample (resulted from pooling of islets extracted from *n* = 7 non-diabetic subjects) and one diabetic sample (resulted from pooling of islets extracted from *n* = 2 diabetic subjects) and OD islets from one individual non-diabetic and one individual diabetic subject are reported in Table 4.

**Figure 2:**
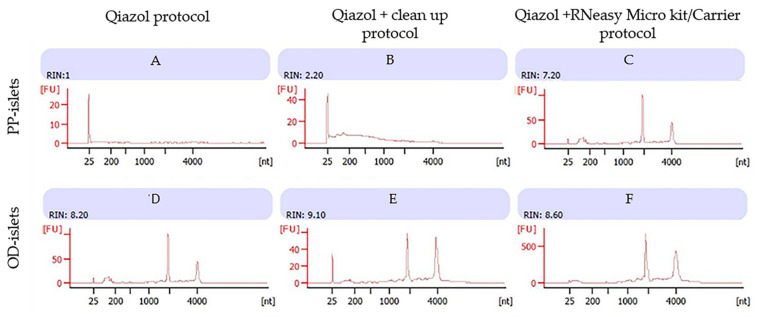
Non-diabetic subjects (one PP sample and one OD sample): Bioanalyzer evaluation of RNA purity obtained by three different protocols: images A and D using Qiazol protocol; images B and E using Qiazol + Clean-up protocol; images C and F using Qiazol + RNeasy Microkit/Carrier protocol. Images labeled (**A**), (**B**) and (**C**) refer to the RNA profiles of human islets from non-diabetic pancreatectomized patients; images labeled (**D**), (**E**) and (**F**) refer to RNA profiles of non-diabetic organ donors; 1 μL of RNA of each sample was applied on a PicoAChip and analyzed for quantity and quality with the Agilent 2100 Bioanalyzer (by Agilent, Santa Clara, CA, USA). FU: fluorescence units, nt: nucleotides. The RNA concentrations (pg/µL) of the samples were (1) 19, (2) 878, (3) 100, (4) 1.33, (5) 12, and (6) 15.24.

Using the Qiazol protocol, we observed a consistent RNA concentration in the PP islet LCM samples from the non-diabetic subject (Table 4), while the bioanalyzer assessment showed low RIN values probably due to ethanol contamination and inadequate washing of pellets (Figure 2A). However, this effect was not confirmed on islets from the non-diabetic OD, in which despite a low concentration (1.33 pg/µL), we reported a high RNA quality with RIN = 8.6 (Figure 2D).

The use of silica membrane columns in the Qiazol/Clean-up protocol reduced ethanol contamination, leading to increased RNA quality in PP islets of non-diabetic subjects (Figure 2B). This effect was also confirmed when the protocol was applied to non-diabetic OD samples (Figure 2E).

Furthermore, the Microkit/Carrier protocol resulted in an improvement in RNA quality in non-diabetic PP islets (Figure 2C). A high RIN was also reported in non-diabetic OD samples (Figure 2F).

We also compared the last three protocols with Qiazol (for convenience named Qiazol, Qiazol/Clean-up, and Microkit/Carrier) on LCM islets of diabetic PPs as well as on handpicked islets of diabetic ODs.

**Figure 3:**
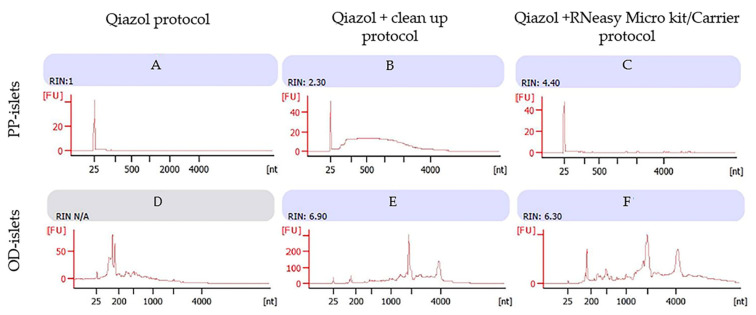
Diabetic subjects (one PP sample and one OD sample): Bioanalyzer evaluation of RNA purity obtained by three different protocols: images A and D using Qiazol protocol; images B and E using Qiazol + Clean-up protocol; images C and F using Qiazol + RNeasy Microkit/Carrier protocol. Images labeled (**A**), (**B**) and (**C**) refer to RNA profiles of human islets from non-diabetic pancreatectomized patients; images labeled (**D**), (**E**) and (**F**) refer to RNA profiles of non-diabetic organ donors; 1 μL of RNA of each sample was applied on a PicoAChip and analyzed for quantity and quality with the Agilent 2100 Bioanalyzer (by Agilent, Santa Clara, CA, USA). FU: fluorescence units, nt: nucleotides. The RNA concentrations (pg/µL) for the samples were (1) 42, (2) 1.96, (3) 29, (4) 125, (5) 25, and (6) 17.88.

Using the Qiazol protocol, we observed a consistent RNA concentration in the LCM samples from diabetic PPs, while the bioanalyzer assessment showed RIN = 1 (Figure 3A). In the diabetic OD sample, we were unable to detect RNA quality (RIN = N/A), probably due to sample contamination (Figure 3D).

The use of silica membrane columns in the Qiazol/Clean-up protocol resulted in an improvement of RNA quality for diabetic PP islets (Figure 3B), which was also confirmed in diabetic OD islets samples (Figure 3E). These results were comparable to the RNA quality detected in non-diabetic subjects (both PPs and ODs).

The Microkit/Carrier protocol showed a consistent improvement of the RNA integrity number in RNA from diabetic PP islets (Figure 3C), while no improvement was detected in the RIN in diabetic OD islets (Figure 3F).

The table summarizing and comparing bioanalyzer results of RNA extracted using the three protocols with Qiazol from islet samples of PP and OD subjects is reported in Supplementary Data (Table S1).

## 5. Discussion

In this study, we aimed to identify an optimal protocol to recover high-quality RNA from human islets isolated by LCM that can be used for next-generation transcriptomic analysis. Our results suggest that the Microkit/Carrier protocol results in improved RNA quality from islets collected by LCM from both non-diabetic as well as diabetic subjects. All experiments were performed on freshly isolated pancreas samples from living patients undergoing partial pancreatectomy. These are extremely valuable tissues, and we believe that pancreatic surgery provides a rare opportunity to correlate in vivo endocrine and metabolic pathways (before surgery) with ex vivo data generated after collecting the pancreatic samples [16]

Previous reports on cell culture and peripheral blood mononuclear cells (PBMCs) have reported that silica gel column technology using the RNeasy Mini-/Microkit is superior in its ability to extract high-quality RNA compared to protocols using the traditional guanidine isothiocyanate extraction method [17], which is mostly explained by the high binding power of silica gel columns. There also appears to be a different outcome in terms of RNA quantity and quality between the two methods. For example, while the guanidine thiocyanate (GTC) technique yields a higher amount of RNA compared to the approach with the silica gel column (SGC), the RNA integrity of fresh as well as frozen samples of lung tissue appears to be improved with the latter method [18]. This relative loss of RNA quantity with the SGC technique could be related to the use of columns holding RNA molecules that are only ~200 nt. The loss of long non-coding RNA (more than 200 nt) by using the Microkit method could represent a disadvantage by limiting important insights into molecular functions provided by large-scale sequencing studies [19].

Despite some limitations, such as the tedious nature of the process and costs, laser capture microdissection (LCM) has several advantages, e.g., minimizing contamination from neighboring cells and avoiding changes due to hypoxia-induced stress that occurs during the normal islet isolation process. Therefore, LCM remains a suitable approach to extract RNA/protein for studies aimed at gene and protein expression in targeted cells. Furthermore, the LCM technique guarantees isolation of high-quality RNA from specific cells from frozen samples. This is especially important when considering formalin-fixed tissues that typically provide low yields and poor quality of extractable DNA and RNA [20], in part due to formalin-induced modifications of the structure and chemistry of nucleic acids, particularly RNA [21].

Gene and protein expression using DNA microarrays, high-throughput sequencing, or proteomics analyses comparing various physiological or experimental conditions represents an essential tool in diverse biological fields to decipher mechanisms and to identify novel signaling pathways. In the islet biology field, RNA sequencing technologies have a high likelihood of identifying novel islet cell-specific promoters or splice forms as well as long non-coding RNAs, which cannot be detected by array analysis. Successful next-generation sequencing analyses require RNA samples free of contamination and degradation, easily obtained for sundry tissues using conventional protocols for RNA preparation. However, RNA isolation from pancreas samples continues to represent a challenge, given the high levels of endogenous RNases, DNases, and proteases that can quickly induce cell autolysis upon dissection. Consequently, classical RNA extraction protocols based on the phenol guanidine thiocyanate method yield RNAs of insufficient quality for subsequent sequencing analysis. In a published protocol for LCM of human islets from surgical specimens, the mean value of the RNA integrity number obtained was 5.8 [13], while in a more recent study, the authors reported an RNA integrity number mean value of 6 for human pancreatic islets derived from healthy brain-dead donors after adding an RNase inhibitor [12].

In our experience, Nanodrop results showed no correlation with effective RNA purity, and we emphasize the importance of bioanalyzer assays to reliably evaluate the yield and quality of RNA. Indeed, it is known that 260/280 absorbance could be affected by variables such as pH, phenol or alcohol contamination, or the presence of proteins [22,23]. Moreover, by analyzing surgical samples obtained from live subjects [24,25,26], instead of brain-dead donors, we minimized the interference from the effects of ischemia and the activation of subsequent inflammatory processes on gene expression [27,28].

Unlike previous studies wherein gene expression is evaluated in beta cell samples enriched by cell fluorescence, our approach used RNA from beta cells that continue to be in the islet microenvironment until extraction and therefore reflects interactions between the different endocrine cell types that is lacking in the former method.

In summary, our comparative studies indicate that the Microkit/Carrier protocol provides a relatively better yield in both the quality and the quantity of RNA extracted from human pancreatic samples compared to the other methods, and we propose this approach for studying differences in gene expression between groups.

## Figures and Tables

**Table 1:** Subject characteristics prior to surgery, classified into non-diabetic and diabetic groups according to an oral glucose tolerance test.

Characteristics	Non-Diabetic (*n* = 7)	Diabetic (*n* = 2)	*p*-Value
Age (years)	57.2 ± 18.8	62.1 ± 22.1	0.70
Sex (F/M)	6/1	1/1	
Body mass index (kg/m^2^)	24.4 ± 5.76	30.5 ± 1.34	0.20
Fasting glucose (mg/dL)	81.6 ± 16.8	135±16.6	**0.006**
2 h OGTT glucose (mg/dL)	109±21.5	225± 7.07	**0.008**
Fasting insulin (mUI/mL)	3.90 ± 2.41	9.95 ± 4.01	0.05
2 h OGTT insulin(mUI/mL)	20.3 ± 15.7	56.3 ± 6.17	0.17
Matsuda index	8.45 ± 2.73	5.33 ± 2.44	0.26
Triglycerides (mg/dL)	101 ± 37.6	95.5± 26.2	0.83
Total cholesterol (mg/dL)	182 ± 25.5	185 ± 20.6	0.94
Cholesterol LDL (mg/dL)	134 ± 19.2	126 ± 30.3	0.83
HbA1c (mmol/mol)	30.0 ± 6.95	40.5 ± 6.36	0.11

Variables are expressed as the mean value ± SD; *p*-value interaction between the two groups, (bold text indicates a statistically significant difference with a *p*-value less than 0.05).

**Table 2:** Nanodrop assessment of RNA samples extracted with the Arcturus PicoPure extraction kit before and after the purification step with the RNeasy Mini Clean up kit in PP islets.

	Before Purification		After Purification	
ID	ng/µL *	260/280	ng/µL ^¶^	260/280
Non-diabetic				
Sample 01	23.59	1.45	12.34	1.59
Sample 02	9.40	1.57	16.73	1.55
Sample 03	25.05	1.45	12.66	1.47
Sample 04	1.94	1.53	2.47	2.60
Sample 05	3.85	0.97	2.85	2.15
Sample 06	35.97	1.23	13.51	1.45
Sample 07	45.40	1.35	10.41	2.57
Mean value ± SD	20.74 ± 16.5	1.36 ± 0.20	10.13 ± 5.44	1.91 ± 0.51
Diabetic				
Sample 01	15.28	1.49	9.29	1.56
Sample 02	31.60	1.36	5.43	2.23
Mean value ± SD	23.44 ± 11.5	1.42 ± 0.09	10.79 ± 2.15	1.89 ± 0.47

Concentration expressed as ng/µL (* 200 µL of total volume, ^¶^ 30 µL of total volume) and 260/280 absorbance of RNA extracted from non-diabetic PP islets (green) and diabetic PP islets (orange) PP: pancreatectomized patients.

**Table 3:** Bioanalyzer evaluation of RNA extracted with the Arcturus PicoPure kit and the RNeasy Mini Clean up Kit in PP islets.

Sample	Bio. Conc. (pg/µL)	Final Bio. Conc. (ng/µL)	RIN
ND_01	150	0.075	1
ND_02	147	0.0735	1
ND_03	93	0.0465	1
ND_04	655	0.3275	1.9
ND_05	533	0.2665	2.5
ND_06	988	0.494	2.6
ND_07	45	0.0225	3.1
Mean value ± SD	373 ± 358	0.18 ± 0.17	1.87 ± 0.89
DM_01	233	0.1165	2
DM_02	96	0.048	1.1
Mean value ± SD	164 ± 96.9	0.08 ± 0.04	1.55 ± 0.64
Pico RNA Bioanalysis Control			10

ND: non-diabetic subjects (green); DM: diabetic subjects (orange); 1 µL of RNA loaded for each sample was applied on Agilent PicoAChip and analyzed for quantity and quality with the Agilent 2100 Bioanalyzer. PP: pancreatectomized patients.

**Table 4:** Nanodrop assessment of RNA samples extracted with the three different protocols using Qiazol as lysis buffer from PP and OD islets.

Sample ID	Protocol	ng/µL	260/280
PP islets: non-diabetic	Qiazol *	23.19	1.37
	Qiazol/Clean-up ^¶^	45.61	1.36
	Microkit/Carrier ^¶^	20.68	1.48
OD islets: non-diabetic	Qiazol *	33.9	1.73
	Qiazol/Clean-up ^¶^	7.61	1.43
	Microkit/Carrier ^¶^	23.85	1.86
PP islets: diabetic	Qiazol *	23.95	1.80
	Qiazol/Clean-up ^¶^	10.63	1.25
	Microkit/Carrier ^¶^	16.24	1.52
OD islets: diabetic	Qiazol *	45.82	1.50
	Qiazol/Clean-up ^¶^	14.31	1.78
	Microkit/Carrier ^¶^	33.45	1.97

Concentration expressed as ng/µL (* 40 µL of total volume, ^¶^ 14 µL of total volume) and 260/280 absorbance of RNA extracted from non-diabetic PP islets (green), non-diabetic OD islets (yellow), diabetic PP islets (orange), and diabetic OD islets (blue). PP: pancreatectomized patient; OD: organ donor.

## Data Availability

The data presented in this study are available on request from the corresponding authors. They take responsibility for the integrity of the data and the accuracy of the data analysis. The data are not publicly available due to privacy issues.

## Supplementary Materials

The following are available online at https://www.mdpi.com/article/10.3390/biom11050625/s1. We inserted a table in Supplementary Materials, which summarizes and compares bioanalyzer results of RNA extracted from islet samples of pancreatectomized patients and organ donors using the three protocols with Qiazol (Table S1).
