# Supplementary material for: A Systematic Comparison of Protocols for Recovery of High-Quality RNA from Human Islets Extracted by Laser Capture Microdissection
Source: Biomolecules. 2021 Apr 22;11(5):625. doi: 10.3390/biom11050625 (PMC8144988; doi:10.3390/biom11050625)
Supplement: Supplementary file 1 [file biomolecules-11-00625-s001.zip › biomolecules-1189816-supplementary.pdf]

biomolecules

Supplementary materials

A Systematic Comparison of Protocols for Recovery of High Quality RNA From Human Islets Extracted by Laser Capture Microdissection

Chiara M. A. Cefalo [1,2,3], Teresa Mezza [1,2,3], Andrea Giaccari [2,3] and Rohit N. Kulkarni [1,*]

[1] Islet Cell Biology & Regenerative Medicine, Joslin Diabetes Center, Department of Medicine, Brigham and Women's Hospital, Harvard Medical School, Harvard Stem Cell Institute, Boston, MA, USA; cefalo.chiara@gmail.com
[2] Dipartimento di Scienze Mediche e Chirurgiche; Centro per le Malattie Endocrino-metaboliche, Fondazione Policlinico Universitario Agostino Gemelli IRCCS, Roma, Italia; teresa.mezza@gmail.com
[3] Dipartimento di Medicina e Chirurgia Traslazionale, Università Cattolica del Sacro Cuore, Roma, Italia; andrea.giaccari@unicatt.it
* Correspondence: Rohit.Kulkarni@joslin.harvard.edu

Citation: Cefalo, C.M.A.; Mezza, T.; Giaccari, A.; Kulkarni, R.N. A Systematic Comparison of Protocols for Recovery of High-Quality RNA from Human Islets Extracted by Laser Capture Microdissection. *Biomolecules* **2021**, *11*, 625. https://doi.org/10.3390/biom11050625

Academic Editor: Roberta Malaguarnera

Received: 2 April 2021
Accepted: 20 April 2021
Published: 22 April 2021

Publisher's Note: MDPI stays neutral with regard to jurisdictional claims in published maps and institutional affiliations.

Copyright: © 2021 by the authors. Licensee MDPI, Basel, Switzerland. This article is an open access article distributed under the terms and conditions of the Creative Commons Attribution (CC BY) license (http://creativecommons.org/licenses/by/4.0/).

Table S1. Bioanalyzer assessment of RNA samples extracted using three different protocols with Qiazol from non-diabetic or diabetic patients following partial pancreatectomy (PP) and islets from organ donors (OD).

SAMPLE ID	PROTOCOL	RIN number	Bio. Conc. (pg/ µl)
PP islets: non-diabetic	QIAZOL	1	19
	QIAZOL/CLEAN UP	2.2	878
	MICROKIT/CARRIER	7.2	100
OD islets: non-diabetic	QIAZOL	8.20	1.33
	QIAZOL/CLEAN UP	9.10	12
	MICROKIT/CARRIER	8.60	15.24
PP islets: diabetic	QIAZOL	1	42.2
	QIAZOL/CLEAN UP	2.2	1.96
	MICROKIT/CARRIER	4.4	29.4
OD islets: diabetic	QIAZOL	N/A	125
	QIAZOL/CLEAN UP	6.9	25
	IMICROKIT/CARRIER	6.3	17.88

One ul RNA loaded for each sample was applied to AgilentPicoAChip and analyzed for quantity and quality with the Agilent 2100 Bioanalyzer. PP: pancreatectomized patients. OD: organ donor patients.
